# Characterization of Postural Sway in Women with Osteoporosis and a Control Group by Means of Linear and Nonlinear Methods

**DOI:** 10.3390/bioengineering10040403

**Published:** 2023-03-24

**Authors:** Felix Stief, Anna Sohn, Lutz Vogt, Andrea Meurer, Marietta Kirchner

**Affiliations:** 1 Department of Orthopedics (Friedrichsheim), University Hospital, Goethe University Frankfurt, 60590 Frankfurt am Main, Germany; 2 Dr. Rolf M. Schwiete Research Unit for Osteoarthritis, Department of Orthopedics (Friedrichsheim), University Hospital, Goethe University Frankfurt, 60590 Frankfurt am Main, Germany; 3 Department of Sports Medicine and Exercise Physiology, Institute of Sports Sciences, Goethe University Frankfurt, 60487 Frankfurt am Main, Germany; 4 Institute of Medical Biometry, University Hospital Heidelberg, 69120 Heidelberg, Germany

**Keywords:** postural control, osteoporosis, risk of falls, center of pressure, variability, multiscale entropy, wavelet

## Abstract

The mechanisms underlying the altered postural control and risk of falling in patients with osteoporosis are not yet fully understood. The aim of the present study was to investigate postural sway in women with osteoporosis and a control group. The postural sway of 41 women with osteoporosis (17 fallers and 24 non-fallers) and 19 healthy controls was measured in a static standing task with a force plate. The amount of sway was characterized by traditional (linear) center-of-pressure (COP) parameters. Structural (nonlinear) COP methods include spectral analysis by means of a 12-level wavelet transform and a regularity analysis via multiscale entropy (MSE) with determination of the complexity index. Patients showed increased body sway in the medial–lateral (ML) direction (standard deviation in mm: 2.63 ± 1.00 vs. 2.00 ± 0.58, *p* = 0.021; range of motion in mm: 15.33 ± 5.58 vs. 10.86 ± 3.14, *p* = 0.002) and more irregular sway in the anterior–posterior (AP) direction (complexity index: 13.75 ± 2.19 vs. 11.18 ± 4.44, *p* = 0.027) relative to controls. Fallers showed higher-frequency responses than non-fallers in the AP direction. Thus, postural sway is differently affected by osteoporosis in the ML and AP directions. Clinically, effective assessment and rehabilitation of balance disorders can benefit from an extended analysis of postural control with nonlinear methods, which may also contribute to the improvement of risk profiles or a screening tool for the identification of high-risk fallers, thereby prevent fractures in women with osteoporosis.

## 1. Introduction

Osteoporosis is a skeletal disorder characterized by low bone mass leading to increased bone fragility and a rapid rise in the risk of sustaining fractures from falls [1,2]. The most consistent predictors of future falls are clinically detected variations in gait or balance [3]. Regarding gait parameters, spatiotemporal parameters (i.e., walking speed and stride length), maximum knee flexion during the swing phase, and ankle power generation during push-off seem to be useful to differentiate between fallers and non-fallers in women with osteoporosis [4]. In this study, fallers were found to walk with a decreased walking speed, stride length, and knee flexion during the swing phase and with reduced ankle power generation during push-off. Force platform measurement is also widely applied to evaluate balance performance and to predict falls among the elderly [5,6,7]. Increased displacement of the center of pressure (COP) indicates poor balance and a higher risk of falls [8,9]. Medial–lateral (ML) body sway seems to underlie age-related changes [10,11,12]. In addition, increased anterior–posterior (AP) body sway has been proposed to be associated with one or more falls [13]. Concerning postural control in patients with osteoporosis, Kuczynski and Ostrowska [14] found increased ML postural sway in women with osteoporosis compared to values obtained from healthy controls reported in the literature. Because no control group was included, a direct comparison was not provided. Abreu et al. [11] found greater oscillations for osteoporotic women, suggesting worse balance compared to controls. Furthermore, Bhattacharya et al. [15] showed that patients with osteoporosis and vertebral fractures have larger postural sway excursions in the ML and AP directions. Hyperkyphosis in patients with osteoporosis is suggested as the leading cause of sagittal plane deformity and seems to be associated with poor balance [16,17]. These linear methods quantify the magnitude of body sway under the assumption that larger variability indicates poor balance.

Notably, in recent years, the limitations of traditional (linear) posturographic methods (e.g., range of motion) on which most postural control studies are based have been discussed [18,19,20]. It has been shown that the quantification of dynamical properties of postural fluctuations (structure of variability) is sufficiently sensitive to identify disease states [18] and fall risk [21]. Stergiou and Decker [22] remarked that the two perspectives of movement variability have to be considered as complementary since each captures different characteristics of the signal.

However, there is a lack of research regarding the characteristics of these parameters in women with osteoporosis compared to a control group of the same age. Previous studies on the impact of osteoporosis on the ability to balance showed general trends [23]. However, studies differ with respect to additional illnesses of the patients. Therefore, the aim of the present investigation was to specifically reveal the impact of osteoporosis at an advanced disease stage in patients without coexisting skeletal and neurological comorbidities, such as fractures and misalignments. Thus, the present explorative study was designed to investigate differences in balance performance between women with osteoporosis and healthy female controls of the same age with the primary aim of identifying COP parameters that differentiate between the two groups. We hypothesized that the two groups differ with respect to the amount of sway, as measured by traditional linear COP parameters, as well as with respect to the structure of sway, as measured by nonlinear COP parameters. As a secondary analysis, osteoporotic women with (fallers) and without (non-fallers) a fall incident were compared with respect to the amount and structure of sway. In this way, we aimed to gain further insight into the mechanisms that may contribute to an increased fall risk in women with osteoporosis, which is helpful to derive potentially preventive exercise concepts.

## 2. Materials and Methods

### 2.1. Participants

Fifty women with osteoporosis who had been referred for bone mineral density (BMD) testing (dual-energy X-ray absorptiometry, DXA) and 25 healthy female control subjects of the same age without osteoporosis and without a fall incident were screened for the present study. Subjects were recruited using flyers and through ambulant consultation hours at our hospital. According to the World Health Organization criteria, osteoporosis was defined as a BMD value of at least 2.5 standard deviations below a mean healthy reference population (T-score ≤ −2.5 by DXA at the lumbar spine or proximal femur or both) [24].

All participants were screened before study enrollment based on a self-administered medical history questionnaire. In addition, a clinical examination was performed in each participant to exclude any other concomitant orthopedic diseases. Patients were asked about an unintentional fall on the ground, floor, or other lower level during walking for a period of one year. One or more falls were assessed by self-report using a fall-assessment questionnaire. The validity of this procedure was evaluated against a calendar-reported method by Mackenzie et al. [25] (84% agreement, 56% sensitivity). On the day of balance testing, all subjects completed the long version of the International Physical Activity Questionnaire (IPAQ) [26] to assess individual physical activity (Table 1). The long form, a seven-day recall of physical activities, asks details about the specific types of activities undertaken within each of the following four domains: leisure time physical activity, domestic and gardening (yard) activities, work-related physical activity, and transport-related physical activity. Data obtained from the IPAQ were computed for the metabolic equivalent of task (MET) minutes spent on moderate to vigorous activity per week. MET is a common physiological measure expressing the energy cost (or calories) of physical activities.

Subjects were excluded from participation if they were unable to stand or walk without an assistive device and/or suffered from orthopedic disorders; spinal stenosis; inflammatory rheumatic disease; musculoskeletal pain; neuropathic pain; or cardiopulmonary, musculoskeletal, somatosensory, psychiatric, or neurological disorders associated with a high risk of falls (e.g., Parkinson’s disease, stroke, muscular dystrophy, epilepsy, Alzheimer’s disease, or cerebral palsy). Subjects were also excluded if they had severe visual and vestibular loss, ophthalmic disorders, morbid obesity with a body mass index ≥30, surgery within the last 12 months, endoprosthetic care, or leg length discrepancy of more than 1 cm or if they were taking medications associated with an increased risk of falling, such as hypnotics, antiepileptics, or antidepressants. Finally, 41 patients (17 fallers and 24 non-fallers) and 19 controls met the inclusions criteria. Participants were thoroughly familiarized with the study design before giving informed consent to participate, as approved by the local ethics committee (319/11) and in accordance with the Declaration of Helsinki.

### 2.2. Center-of-Pressure Recording and Data Analyses

One AMTI force plate (Advanced Mechanical Technology, Inc., Watertown, MA, USA) was used to collect ML and AP displacement data of the COP at a sampling rate of 200 Hz. Subjects stood with both legs and bare feet on the force plate. The feet were shoulder width apart and pointing forward. All subjects were asked to maintain this stance as still as possible for 60 s with arms akimbo in a neutral position, facing straight ahead and eyes closed. An eyes-closed assessment protocol was chosen because we expected that this approach is more “provocative” and has a greater effect on equilibrium regulation and postural control compared to an eyes-open protocol. It has also been shown that eye closure destabilizes posture, resulting in a significant increase in body weight distribution asymmetry in the elderly [27]. Three trails were conducted for each subject. From the initial 60 s of each balance measurement, the first and the last 15 s were extracted, and only the middle 30 s were evaluated and processed. Signal preprocessing included detrending and downsampling to 100 Hz [28,29].

The amount of sway was characterized by traditional (linear) COP parameters, i.e., temporal (standard deviation (SD, mm), range of motion (ROM, mm), mean velocity (V, mm/s)), spatiotemporal (path length (PL, mm²)), and frequency (f80, Hz) parameters, in order to cover different aspects of sway characteristics. To compute f80, a power spectral density (PSD) based on Welch’s algorithm [30,31] was used, and the frequency below which 80% of the total power occurred was determined. The 80th percentile was chosen as it is suggested to best characterize the modifications of the postural control system [32]. More details on the computation of the mentioned parameters can be found in Appendix A (Table A1).

Structural (nonlinear) COP methods included a spectral analysis by means of a 12-level wavelet transform (WT) and a regularity analysis via multiscale entropy (MSE) with determination of the complexity index (CI), which is the area under the MSE curve [33]. WT outputs energy as a percentage of the total energy for different frequency bands [34]. It can highlight the intermittent activity of neuromuscular feedback loops at different time scales [35,36,37]. MSE was applied to analyze the regularity and complexity of the COP signal. It outputs sample entropy (SaEn), which grows monotonically with the degree of randomness for each scale. More details, including a table with relevant input parameters, can be found in Appendix A and Appendix B. Linear and nonlinear methods were applied to each trial using MATLAB software Version R2020b (The MathWorks, Inc., Natick, MA, USA). The mean of the three trials per subject was used for further statistical analyses.

### 2.3. Statistical Analysis

Normal distribution was checked by the Shapiro–Wilk test (α = 0.05). In the case of normally distributed data, unpaired *t*-tests were applied (otherwise, nonparametric Mann–Whitney U-tests) to test for a group effect (primary analysis: patients vs. controls; secondary analysis: fallers vs. non-fallers) for each linear COP parameter and for the CI at a significance level of α = 0.05 (two-sided). In addition, unpaired *t*-tests or Mann–Whitney U-tests were used to assess whether the groups differed with respect to anthropometric parameters (age, body height, body mass, and body mass index). Concerning MSE and WT, 95% confidence intervals were calculated for each scale or level.

This is an explorative analysis. No α-adjustments for multiple testing were applied. Descriptive *p*-values ≤ 0.05 were considered statistically significant. Statistical analysis was performed in SPSS version 21 (IBM Corporation, Armonk, NY, USA).

## 3. Results

Normality of the data could only be assumed for body height, SD (AP), f80 (ML), and CI (ML) according to the results of the Shapiro–Wilk test. Descriptive results (mean ± standard deviation) of anthropometric parameters, BMD value, and the IPAQ stratified by group are shown in Table 1. No significant differences (*p* > 0.05) were found between patients and controls or between fallers and non-fallers for any anthropometric parameters, T-score, or self-reported physical activity assessed with the IPAQ.

### 3.1. Primary Analysis: Patients vs. Controls

Table 2 presents the mean ± standard deviation and the results of the test statistics with *p*-values concerning the traditional (linear) COP parameters and the CI. With respect to traditional COP parameters, osteoporotic women showed significantly increased body sway (SD, ROM) in the ML sway direction compared to controls. No significant differences were found between fallers and non-fallers.

**Figure 1:**
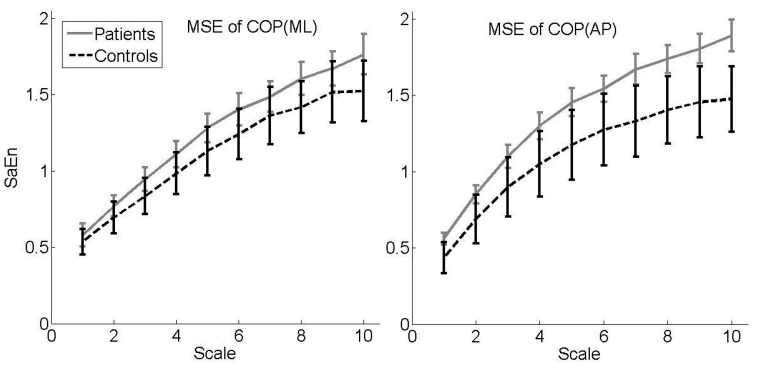
Sample means with 95% confidence intervals of sample entropy (SaEn) by scale *i* = 1, …, 10 for COP (ML) (**left** panel) and COP (AP) (**right** panel) for controls vs. patients. Scale *i* corresponds to a time scale in seconds (s) of $\frac{3i}{20}$ s (e.g., *i* = 1 ≙ 0.15 s). MSE = multiscale entropy, COP = center of pressure, ML = medial–lateral, AP = anterior–posterior.

Figure 1 presents MSE curves (sample mean, 95% confidence interval) with SaEn plotted against scale stratified by group for each COP sway direction. Irrespective of the sway direction, controls had lower values on all scales, with non-overlapping confidence intervals for scales 7 to 10 with respect to the AP sway direction. This is also reflected in the CI, as controls had statistically significant lower values compared to patients for the AP sway direction (Table 2).

**Figure 2:**
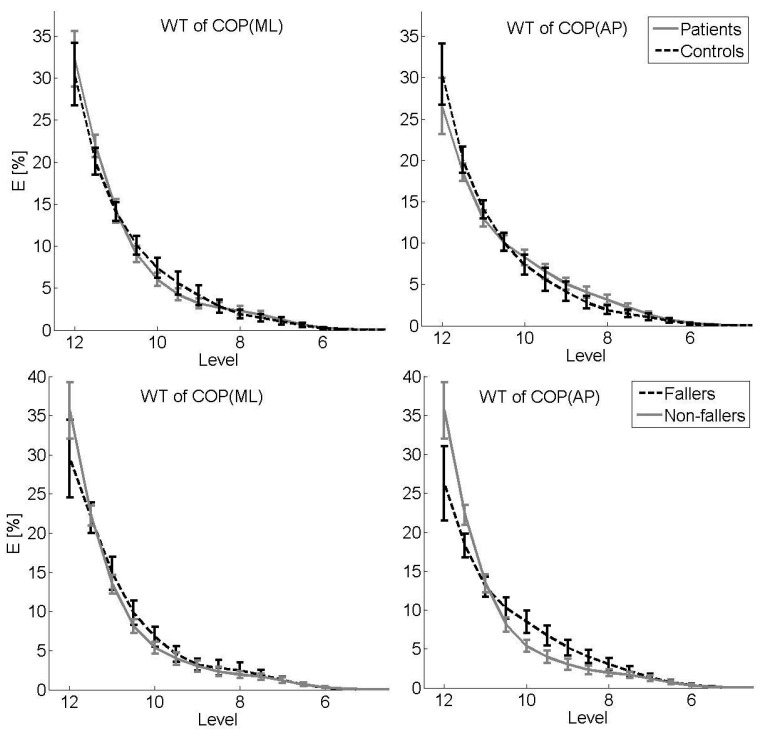
Energy (E) as a percentage of total energy for levels *j* = 12, …, 5 for COP (ML) (**left** panel) and COP (AP) (**right** panel) for controls vs. patients (**upper** panel) and fallers vs. non-fallers (**lower** panel). Sample means with 95% confidence intervals are shown. Level *j* corresponds to a frequency of *f* = 80/2*^j^* Hz, e.g., *j* = 12 ≙ *f* = 0.02 Hz. WT = wavelet transformation, COP = center of pressure, ML = medial–lateral, AP = anterior- posterior.

Figure 2 shows descriptive results of WT (sample mean, 95% confidence interval) stratified by group (upper panel: patients vs. controls) for each COP sway direction. Energy as a percentage of total energy is presented for levels 5 to 12 (corresponding to a frequency range of 2.5 to 0.02 Hz). Concerning the ML sway direction, the sample mean for the control group is larger for the higher-frequency bands (levels 9 to 10.5), with inverse results for the AP sway direction (larger sample mean for patients for levels 7.5 to 10), although with overlapping confidence intervals on all levels.

### 3.2. Secondary Analysis: Fallers vs. Non-Fallers

With respect to traditional COP parameters, no statistically significant differences (*p* > 0.05) were found between fallers and non-fallers (Table 2). MSE analysis revealed similar SaEn values for the two groups on each scale, with no statistically significant differences in the CI, irrespective of the sway direction (Table 2). WT showed more energy in the higher-frequency bands (levels 8 to 10.5) and less energy in the low-frequency bands (levels 11.5 to 12) for fallers compared to non-fallers for the AP sway direction (Figure 2, lower panel).

## 4. Discussion

The aim of the present study was to identify COP parameters that differentiate women with osteoporosis from healthy female controls according to their balance performance. Based on the defined inclusion and exclusion criteria, the present sample reveals the specific impact of osteoporosis without the occurrence of skeletal comorbidities at an advanced disease stage. Strict exclusion criteria led to a very homogenous group of patients and controls, with accordance in demographic data only differing in terms of the diagnosis of osteoporosis through DXA. Group differences could therefore not be attributed to the effect of aging, extent of osteoporosis (T-score), or physical activity. In particular, the results of the IPAQ indicate that fallers did not show decreased physical activity compared with non-fallers. According to Smulders et al. [38], this could be due to increased safety awareness, which may have eliminated any unfavorable effects (e.g., physical inactivity) of a fall incident.

Among the elderly, greater COP displacement measured with a force platform is typically used to indicate poor balance and fall risk [8,9]. Here, we observed larger sway in the ML direction in females with osteoporosis compared to healthy controls in a quiet standing task. This finding indicates limited stability in patients based on the assumption that increased sway variability is equalized with less stability. It has been shown that for AP postural control, a dominant hip load/unload response is present, with negligible contributions by the ankle plantar-/dorsiflexors. On the other hand, ML balance is dominated by the ankle muscles [39]. This could indicate that in women with osteoporosis, the muscles surrounding the ankle joint are particularly negatively affected compared to age-matched subjects without osteoporosis. While previous studies described that postural stability in the ML sway direction is a major problem, especially in the context of falling [40,41,42], no differences in the context of falling could be found in the present study. However, the amount of sway is not usually conclusive evidence of instability, as other proofs are needed in relation to the dynamics of postural control [43]. Hence, a decrease in COP area can be a sign of a better integration of multisensory inputs, as well as a sign of increased body stiffness associated with fear of falling [44]. Thus, we also investigated structural characteristics of the COP signal. Differences between patients and controls were mainly found for the AP sway direction. A more irregular motor output, as expressed by larger SaEn values on several scales, resulting in a larger CI, was found for patients compared to controls. In terms of the postulated relationship between COP regularity and the amount of attention invested [45], this indicates that patients invest less attention to control their posture. As subjects were forced to stand as still as possible, these results indicate that controlling posture is more difficult for patients. This is partly in line with the results of the WT with respect to the AP sway direction, as higher-frequency responses were found for patients, suggesting more frequent postural changes, which can be interpreted as a poor ability to avoid postural sway in the context of a quiet standing task. However, confidence intervals overlap, and with respect to the ML sway direction, controls showed higher-frequency responses. Future studies should focus on this relationship in order to confirm our results.

A comparison of fallers and non-fallers within the patient group revealed no differences in terms of the regularity measures (SaEn and CI from MSE). However, WT showed higher-frequency responses in fallers for the AP sway direction, suggesting difficulties in avoiding postural sway in a quiet standing task.

In summary, in the present study, COP parameters were found to be partly appropriate to differentiate between women with osteoporosis and a healthy control group of the same age. It can be concluded that postural sway in the ML and AP directions are differently affected by osteoporosis. Based on our results, we assume that patients with osteoporosis, regardless of a fall event, can be identified by increased ML displacement in traditional linear balance measurement compared to controls, as well as by more irregular sway patterns in the AP sway direction in a quiet standing task. Osteoporosis is a complex systemic, endocrinologic disease with multifactorial impacts. Therefore, it is difficult to identify a single disturbance variable. Due to demographic changes with an increasingly aging population, a further increase in falls can be expected, particularly in patients with osteoporosis. It is therefore an important objective to identify patients with osteoporosis and an increased fall risk based on linear and nonlinear COP parameters. Thus, the additional mathematical effort for structural, nonlinear methods is appropriate to enhance our understanding of postural control in women with osteoporosis and postural control mechanisms in general.

The results of the present study should be interpreted in the light of its limitations. Although a large group of 50 patients was screened for the present study, the group of fallers was relatively small. In addition, no adjustment for multiple testing was applied; therefore, our results and their interpretation have an exploratory character and should be treated with some caution. Strict exclusion criteria regarding concomitant orthopedic diseases led to a homogenous group of patients with osteoporosis. However, detailed information regarding the status of the sensorimotor function of patients and controls was not available, which may have had an effect on the results of the balance test. A further limitation of the present study is that postural control tested in a quiet standing task with eyes closed alone is often insufficient to reveal the underlying postural control mechanisms. Therefore, the investigation of postural control when standing with eyes open, in dual-task settings, or different positions can help to further understand postural control mechanisms by enabling investigation of adaptations to different conditions and improved interpretation of postural sway characteristics [46,47]. It has been shown that women with osteoporosis have weaker quadricep strength than counterparts with normal bone mass [12]. In addition, weaker quadricep strength seems to be a powerful parameter to distinguish fallers from non-fallers among women with osteoporosis [4]. Therefore, future research should address the association between muscle strength and postural sway characteristics.

## 5. Conclusions

In conclusion, the present study suggests that more than one measure of balance is needed to understand the failure of a system in a given environment. Besides traditional linear methods, structural nonlinear methods are helpful to explain differences in postural control between women with and without osteoporosis and between fallers and non-fallers. We observed larger sway in the ML direction and more irregular sway in the AP direction, as expressed by larger SaEn values on several scales, resulting in a larger CI in females with osteoporosis compared to healthy controls. Moreover, fallers showed higher-frequency responses than non-fallers in the AP direction. Clinically, effective assessment and rehabilitation of balance disorders can benefit from an extensive analysis of postural control, which may also contribute to the improvement of risk profiles or a screening tool for the identification of high-risk fallers, thereby preventing fractures in women with osteoporosis.

## Figures and Tables

**Table 1:** Mean ± standard deviation for anthropometric parameters, T-score, and physical activity for controls and patients (women with osteoporosis), as well as for osteoporotic women with (fallers) and without (non-fallers) a fall incident.

Parameter	Controls (n = 19)	Patients (Women with Osteoporosis)		
		All (n = 41)	Fallers (n = 17)	Non-Fallers (n = 24)
Age (years)	68.3 ± 5.3	70.0 ± 5.4	70.8 ± 4.3	69.4 ± 6.1
Height (m)	1.61 ± 0.05	1.61 ± 0.06	1.62 ± 0.06	1.61 ± 0.07
Body mass (kg)	63.5 ± 10.1	65.1 ± 10.0	68.2 ± 10.0	62.9 ± 9.6
BMI (kg/m^2^)	24.5 ± 3.2	25.1 ± 3.9	26.3 ± 4.0	24.2 ± 3.7
Maximum T-score		−3.28 ± 0.61	−3.44 ± 0.64	−3.19 ± 0.59
IPAQ (MET-min/week)	4593 ± 3908	4100 ± 3264	4545 ± 3687	3855 ± 3104

T-score measured by dual-energy X-ray absorptiometry at the lumbar spine or proximal femur or both; IPAQ = International Physical Activity Questionnaire; MET = metabolic equivalent of task.

**Table 2:** Mean ± standard deviation of center-of-pressure parameters for controls and patients (women with osteoporosis), as well as for osteoporotic women with (fallers) and without (non-fallers) a fall incident. Corresponding test statistics (*t*-test or Mann–Whitney U-test) and *p*-values are presented for patients vs. controls and fallers vs. non-fallers.

Parameter	Controls (n = 19)	Patients (n = 41)	Test Statistic	*p*-Value	Fallers (n = 17)	Non-Fallers (n = 24)	Test Statistic	*p*-Value
SD (ML) (mm)	2.00 ± 0.58	2.63 ± 1.00	U = −2.30	**0.021**	2.62 ± 1.16	2.64 ± 0.19	U = −0.20	0.843
SD (AP) (mm)	4.82 ± 0.86	4.92 ± 1.46	T = 0.34	0.734	5.03 ± 1.37	4.84 ± 1.54	T = −0.40	0.690
ROM (ML)	10.86 ± 3.14	15.33 ± 5.58	U = −3.08	**0.002**	15.11 ± 6.68	15.49 ± 4.80	U = −0.64	0.525
(mm)								
ROM (AP)(mm)	25.39 ± 5.23	28.50 ± 8.26	U = −1.33	0.185	28.85 ± 8.58	28.24 ± 8.19	U = −0.19	0.853
V (ML) (mm/s)	10.79 ± 1.72	11.30 ± 3.00	U = −0.41	0.685	10.62 ± 1.41	11.78 ± 3.69	U = −1.05	0.296
V (AP) (mm/s)	13.83 ± 2.90	14.78 ± 2.90	U = −0.93	0.353	14.76 ± 3.70	14.78 ± 2.26	U = −0.70	0.483
PL (mm²)	1170.8 ± 191.4	1241.3 ± 246.5	U = −0.83	0.404	1209.5 ± 229.5	1263.9 ± 260.2	U = −0.79	0.427
f80 (ML) (Hz)	0.37 ± 0.17	0.42 ± 0.16	T = 0.97	0.335	0.42 ± 0.16	0.42 ± 0.17	T = −0.03	0.976
f80 (AP) (Hz)	0.36 ± 0.23	0.42 ± 0.15	U = −1.80	0.072	0.43 ± 0.18	0.41 ± 0.14	U = −0.32	0.750
CI (ML)	11.24 ± 3.31	12.47 ± 2.62	T = 1.56	0.123	12.45 ± 2.44	12.49 ± 1.78	T = 0.05	0.964
CI (AP)	11.18 ± 4.44	13.75 ± 2.19	U = −2.22	**0.027**	13.59 ± 2.71	13.86 ± 1.78	U = −0.23	0.822

ML = medial–lateral, AP = anterior–posterior, SD = standard deviation, ROM = range of motion, V = mean velocity, PL = path length, f80 = frequency, CI = complexity index. Significant differences are indicated in bold.

## Data Availability

The data presented in this study are available upon request from the corresponding author.

## Appendix A. Input Parameters

**Table A1:** Details and input parameters for the different methods applied to the center-of-pressure signal.

Method	Details and Input Parameter
SD(x) (mm)	$\frac{1}{n-1}\sum (x_{i}-\bar{x})²$, $x_{i}=ith$ data point of signal x, $\bar{x}$= mean value of x
ROM(x) (mm)	max(x) − min(x)
V(x) (mm/s)	$\frac{fs}{n-1}\sum |x_{i}-x_{i+1}|$, $x_{i}=ith$ data point of signal x
PL (mm²)	$\sum \sqrt{{\left(x_{i+1}-x_{i}\right)}^{2}+\left(y_{i+1}-y_{i}\right)²}$ with $x_{i}=ith$ data point of ML sway and $y_{i}=ith$ data point of AP sway
PSDWelch’s method	Hamming window of 2000 samples, 50% overlap, nfft =$\;2^{11}$, fs = 100 Hz
WT	Mother wavelet = Coiflet with central frequency fc=0.8 Hz, levels j=12,…, 5, which corresponds to the frequency range pf f=2.5 Hz $\left(j=5\right)$ to f=0.02 Hz $\left(j=12\right)$, fs = 100 Hz
MSE	Radius r=0.15, m=2, scale i=1,…, 10, fs = 20 Hz (downsampling to 20 Hz)

SD = standard deviation, ROM = range of motion, V = velocity, PL = path length, PSD = power spectral density, WT = wavelet transformation, MSE = multiscale entropy, ML = medial–lateral, AP = anterior–posterior, fs = sampling frequency, f = frequency.

## Appendix B. Wavelet Transformation and Multiscale Entropy

Wavelet transformation outputs the energy of level *j* (for more details, see [34]). Mathematically, the wavelet decomposition is a convolution of the time series with wavelets of different scales (*a*) and translations (*b*). The input signal is considered to be composed of summed elementary wavelets, which are time-localized waveforms, as the amplitude tends toward zero at some limit. Coiflet wavelet functions are appropriate to analyze center-of-pressure (COP) data, as they are most effective at reducing low-frequency distortion [37]. We excluded the high-frequency range, i.e., frequencies (*f*) above the range of interest, by starting with *j* = 5, which corresponds to *f* = 2.5 Hz. The following formula expresses the relation between scale (*a* = 2*^j^*) and frequency [32]: *f_a_* = (*fc* ∙ *fs*)/*a*, where *fc =* 0.8 Hz is the center frequency of the Coiflet wavelet. Low scales correlate with high frequencies, as they compress the wavelet. Multiscale entropy (MSE) outputs sample entropy (SaEn) on different scales (*i*) and is a measure of complexity. The area under the MSE curve is called the complexity index [33]. SaEn shows better relative consistency and is less sensitive to the length of data than other entropy measures [48]. SaEn, which was introduced by Richman and Moorman [49], is the negative natural logarithm of the conditional probability that a signal of length N, which has repeated itself within a tolerance (*r*) for *m* points also repeats itself for *m* + 1 points. The choice of *m* and *r* is related to previous work [33,48]. The COP signal is downsampled to 20 Hz in advance to exclude time scales smaller than 0.15 s ($=\frac{m+1}{20}=\frac{3}{20}\;s).$ In addition, downsampling of the signal is necessary prior to the computation of SaEn, as it reduces collinearities [50].
